# The lncRNA NEAT1 activates Wnt/β-catenin signaling and promotes colorectal cancer progression via interacting with DDX5

**DOI:** 10.1186/s13045-018-0656-7

**Published:** 2018-09-05

**Authors:** Meng Zhang, Weiwei Weng, Qiongyan Zhang, Yong Wu, Shujuan Ni, Cong Tan, Midie Xu, Hui Sun, Chenchen Liu, Ping Wei, Xiang Du

**Affiliations:** 1 Department of Pathology, Fudan University Shanghai Cancer Center, Shanghai, 200032 China; 2 Department of Pathology, Shanghai Medical College, Fudan University, Shanghai, China; 3 Institute of Pathology, Fudan University, Shanghai, China; 4 Department of Oncology, Shanghai Medical College, Fudan University, Shanghai, China; 5 Institutes of Biomedical Sciences, Fudan University, Shanghai, China; 6 Cancer Institute, Fudan University Shanghai Cancer Center, Shanghai, China

**Keywords:** NEAT1, lncRNA, DDX5, β-Catenin, Colorectal cancer

## Abstract

**Background:**

The long noncoding RNA nuclear-enriched abundant transcript 1 (NEAT1) has been reported to be overexpressed in colorectal cancer (CRC). However, its underlying mechanisms in the progression of CRC have not been well studied.

**Methods:**

To investigate the clinical significance of NEAT1, we analyzed its expression levels in a publicly available dataset and in 71 CRC samples from Fudan University Shanghai Cancer Center. Functional assays, including the CCK8, EdU, colony formation, wound healing, and Transwell assays, were used to determine the oncogenic role of NEAT1 in human CRC progression. Furthermore, RNA pull-down, mass spectrometry, RNA immunoprecipitation, and Dual-Luciferase Reporter Assays were used to determine the mechanism of NEAT1 in CRC progression. Animal experiments were used to determine the role of NEAT1 in CRC tumorigenicity and metastasis in vivo.

**Results:**

NEAT1 expression was significantly upregulated in CRC tissues compared with its expression in normal tissues. Altered NEAT1 expression led to marked changes in proliferation, migration, and invasion of CRC cells both in vitro and in vivo. Mechanistically, we found that NEAT1 directly bound to the DDX5 protein, regulated its stability, and sequentially activated Wnt signaling. Our study showed that NEAT1 indirectly activated the Wnt/β-catenin signaling pathway via DDX5 and fulfilled its oncogenic functions in a DDX5-mediated manner. Clinically, concomitant NEAT1 and DDX5 protein levels negatively correlated with the overall survival and disease-free survival of CRC patients.

**Conclusions:**

Our findings indicated that NEAT1 activated Wnt signaling to promote colorectal cancer progression and metastasis. The NEAT1/DDX5/Wnt/β-catenin axis could be a potential therapeutic target of pharmacological strategies.

**Electronic supplementary material:**

The online version of this article (10.1186/s13045-018-0656-7) contains supplementary material, which is available to authorized users.

## Background

Colorectal cancer (CRC) is the third most common cancer and the third leading cause of cancer-related death in men and women in the USA, accounting for one-tenth of all cancer-related deaths in women and men [1]. Treatment regimens for advanced CRC involve combination chemotherapies, which are toxic and largely ineffective but have remained the backbone of treatment over the past decade [2]. Hence, genetic and epigenetic alterations and their underlying mechanisms in CRC should be explored more intensively to discover prognostic biomarkers and therapeutic targets for CRC.

Long noncoding RNAs (lncRNAs) are defined as RNA polymerase II transcripts longer than 200 nucleotides in length that lack a significant protein-coding capacity [3, 4]. Over the past two decades, the biogenesis and functional mechanisms of miRNAs have been extensively elucidated. Nevertheless, the roles of lncRNAs remain unclear. lncRNAs have a great biological significance in the occurrence and progression of cancers because they can interact with cancer stem cells and then affect cancer metastasis and recurrence [5]. lncRNAs potentially act through various mechanisms that may be related to a wide range of subcellular localizations, expression levels, and stability in mammalian cells [6, 7]. As expected, increasing evidence suggests that many lncRNAs fulfill their functions through specific interactions with other cellular factors (proteins, DNA, and other RNA molecules). Hence, finding lncRNA interacting partners is considered a strategy to gain insights into their molecular mechanisms [6].

Many lncRNAs potentially act as scaffolds to bring together different proteins or bridge protein complexes via their protein interaction capabilities [8]. For example, NEAT1 and MALAT1 bind multiple proteins localized to paraspeckles and nuclear speckles, respectively [9–11]. The lncRNA NEAT1 is abnormally upregulated in somatic malignancies and has been found to promote tumor growth in CRC [12–14]. Nevertheless, elucidating the molecular mechanisms underlying the oncogenic functions of NEAT1 requires further effort. Identifying the upstream and downstream targets of NEAT1 will elucidate its critical role in tumor progression.

In most cases, inappropriate activation of the proto-oncoprotein β-catenin is thought to induce tumor formation. Mutations of the tumor suppressors APC and axin, which are found in many colorectal tumors, prevent β-catenin degradation, and phosphorylation [15, 16]. All of these mutations cause both nuclear accumulation of β-catenin, thereby contributing to its ability to bind T cell transcription factors, and upregulation of proto-oncogenes, such as c-myc, Axin2, and cyclin D1 [17–19]. Recently, decreased NEAT1 expression was reported to inhibit Wnt/β-catenin signaling pathway activity. However, the molecular mechanisms underlying this phenomenon are unknown.

In this study, we revealed the key functions of NEAT1 in the proliferation, migration, and invasion of CRC cells both in vitro and in vivo. Notably, we provided evidence that NEAT1 directly bound to DDX5 and enhanced its protein stability. NEAT1 activated the transcriptional activity of β-catenin to promote CRC tumor progression in a DDX5-mediated manner. Clinically, NEAT1 expression was elevated in CRC tissues and positively correlated with DDX5 expression. Taken together, these results suggest that NEAT1 and DDX5 in combination may be valuable prognostic predictors for CRC. Thus, the NEAT1/DDX5/β-catenin axis appears to be a promising target for CRC therapy.

## Methods

Please find the complete Materials and Methods in Additional file 1.

### CRC patient information

Fresh samples were obtained from 71 newly diagnosed CRC patients who underwent no preoperative therapy prior to surgical resection at Fudan University Shanghai Cancer Center (FDUSCC) between 2008 and 2009. This study was approved by the institutional review board of Shanghai Cancer Center. The median follow-up time was 80 months, and the longest follow-up was 87 months.

### Transient and stable transfections

NEAT1 siRNAs (si1: sense 5’-GACCGUGGUUUGUUACUAUdTdT-3′, antisense 5′-AUAGUAACAAACCACGGUCdTdT-3′; si2: sense 5′-GUUGGUCAUUCCUAAAUCUTT-3′, antisense 5′-AGAUUUAGGAAUGACCAACTT-3′), si-DDX5 (sense: 5′-GCAAGUAGCUGCUGAAUAUUU-3′, antisense: 5′-pAUAUUCAGCAGCUACUUGCUU-3′), and a scramble siRNA were purchased from GenePharma (Shanghai, China). ShNEAT1 lentiviruses (5′-GACCGUGGUUUGUUACUAU-3′) and shNC (representing the sh-negative control, 5′-UUCUCCGAACGUGUCACGU-3′) were generated by Sbo-Bio (Shanghai, China). Transient transfection of pc3.1-NEAT1, pc3.1 (control vector), or siRNA was performed using Lipofectamine 3000 (Invitrogen, CA, USA) according to the manufacturer’s instructions. To establish stable cell lines, shNEAT1 lentiviruses were transduced into HCT116 and SW1116 cells with polybrene (5 μg/mL; Sigma-Aldrich, MO, USA). Then, the cells were selected with 0.5 μg/mL of puromycin for 14 days. The transfection efficiencies were verified by RT-qPCR and western blotting.

### Animal experiments

This study complied with the Animal Care guidelines of FDUSCC. Male BALB/c nude mice (6 weeks old) were housed under specific pathogen-free conditions. HCT116-shNC and HCT116-shNEAT1 cells were injected either subcutaneously (*n* = 4, 5 × 10^6^/mouse) or into the tail vein (*n* = 4, 2 × 10^6^/mouse). The mice were sacrificed after 4–6 weeks. The tumors and lungs of the mice were removed, fixed in 10% formalin, and stored at − 80 °C for the subsequent analyses. Each animal experiment was performed in triplicate.

### Western blotting

The standard western blotting assay was performed as previously described [20]. The specific primary antibodies are listed in Additional file 2: Table S1.

### RNA pull-down, mass spectrometry, and RNA immunoprecipitation assays

RNA pull-down was performed using the Magnetic RNA-Protein Pull-Down kit (Pierce, MA, USA) in accordance with the manufacturer’s instructions. The protein bands on the gel were silver-stained. Bands of interest were identified by mass spectrometry (MS) and confirmed by western blotting. RNA immunoprecipitation (RIP) was performed using the Magna RIP RNA-Binding Protein Immunoprecipitation Kit (Millipore, MA, USA) according to the manufacturer’s protocol.

### Promoter reporter and Dual-Luciferase Assay

The DDX5 promoter was cloned into the pGL3 basic luciferase reporter vectors (Promega, USA). In total, 5000 cells were seeded into each well of a 96-well plate and transfected with 100 ng of the TOP/FOP-flash reporter plasmids (Millipore, MA, USA), 100 ng of an expression vector (pGL3-DDX5 or pGL3-Basic) or 0.25 μl of siRNA. DDX5 promoter activity and TOP/FOP-flash were normalized by cotransfection with 10 ng of a Renilla luciferase reporter. After 24 h of incubation, the luciferase activity was detected using the Dual-Luciferase Reporter Assay System (Promega, USA).

### Reproducibility

Each experiment was independently repeated at least three times, and the data were presented as the mean ± SD. For the western blotting, EdU, wound healing, RIP, and Transwell assays, the representative results of three independent experiments are shown.

### Statistical analysis

Comparisons between groups were analyzed using Student’s *t* tests for the mRNA levels, and clinicopathological parameters were compared using the *χ*^2^ test. Correlations between the mRNA levels were calculated with Spearman’s rank correlation coefficients. Survival curves were plotted using the Kaplan-Meier method and compared using the log-rank test. *p* < 0.05 was considered significant. All statistical analyses were conducted using the SPSS 19.0 statistical software (SPSS Inc., IL, USA).

## Results

### NEAT1 is upregulated in human CRC tissues and is associated with a poor prognosis in CRC patients

**Fig. 1:**
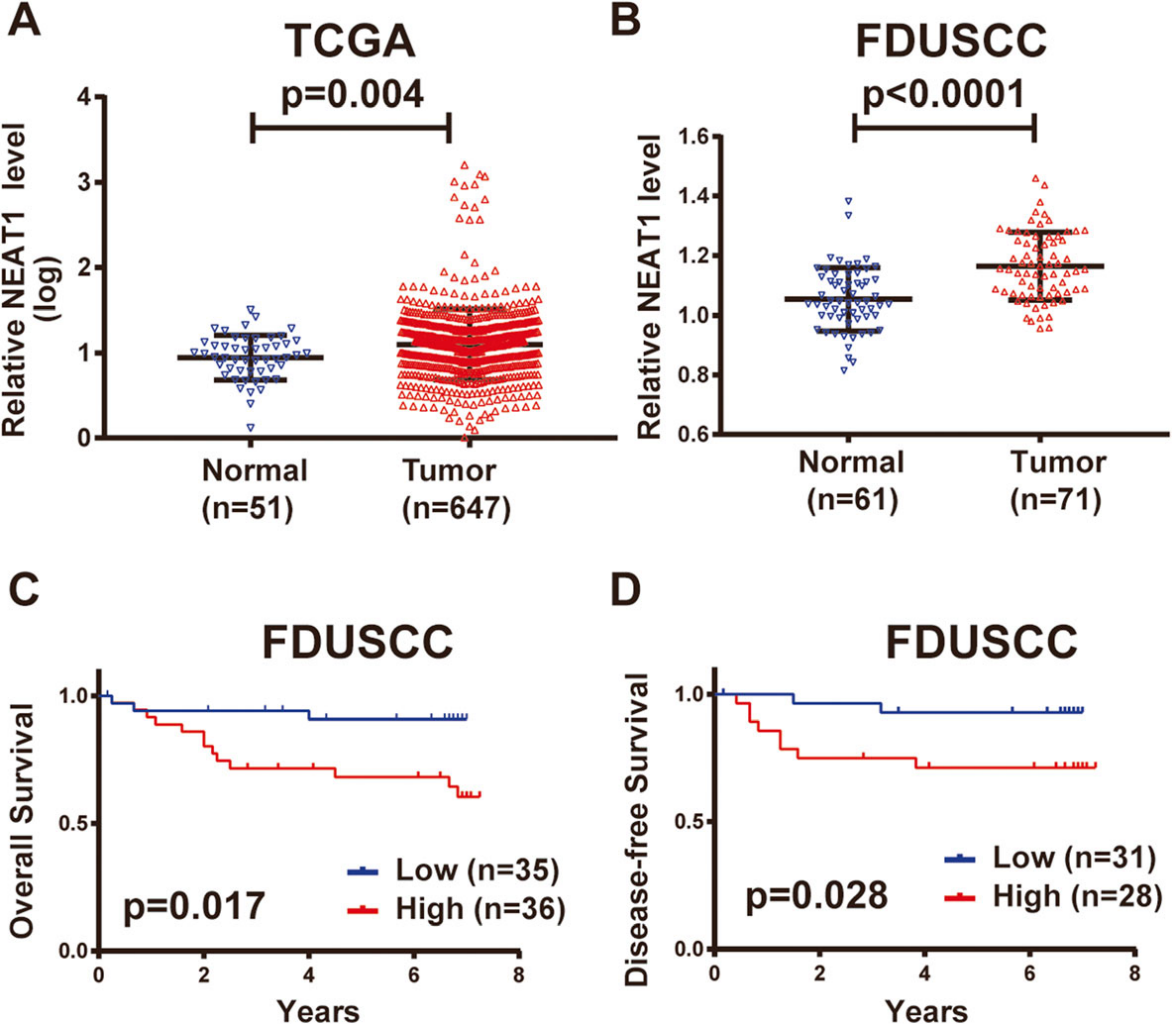
NEAT1 is upregulated in human CRC patients and predicts a poor prognosis. **a** NEAT1 expression in TCGA CRC RNA-seq dataset (normal *n* = 51, tumor *n* = 647). **b** NEAT1 expression in the FDUSCC dataset (normal *n* = 61, tumor *n* = 71). **c**–**d** Kaplan-Meier analyses of the FDUSCC dataset. **c** High NEAT1 expression predicted shorter OS than that of patients with low NEAT1 expression. **d** High NEAT1 expression predicted shorter DFS than that of patients with low NEAT1 expression

To investigate the clinical significance of NEAT1, we analyzed its expression levels in the publicly available TCGA dataset and in data from 71 CRC samples from FDUSCC. Both datasets showed that NEAT1 expression was significantly upregulated in the tumor tissues compared to the levels in the normal tissues (Fig. 1a, b). Correlations between the clinicopathological features of the CRC patients and the NEAT1 levels are summarized in Additional file 2: Table S3. NEAT1 was not related to age, gender, AJCC stage, T stage, N stage, and other features. The univariate Cox proportional hazards models for overall survival (OS) and disease-free survival (DFS) are summarized in Additional file 2: Table S4. Our results revealed that patients with high NEAT1 expression (divided by the mean value) showed obviously poorer OS than those with low NEAT1 expression [23/35 (65.7%) vs. 32/36 (88.9%), HR 4.457, 95% CI 1.267–15.685, *p* = 0.017, Fig. 1c]. In addition, 8 of the 28 (28.6%) patients with high NEAT1 expression experienced recurrence, whereas only 2 of the 31 (6.452%) patients with low NEAT1 relapsed. The Kaplan-Meier curves showed that patients with high NEAT1 expression has poorer DFS than those with relatively low NEAT1 expression (*p* = 0.028, Fig. 1d).

### NEAT1 mediates cell proliferation in vitro and tumorigenicity in vivo

**Fig. 2:**
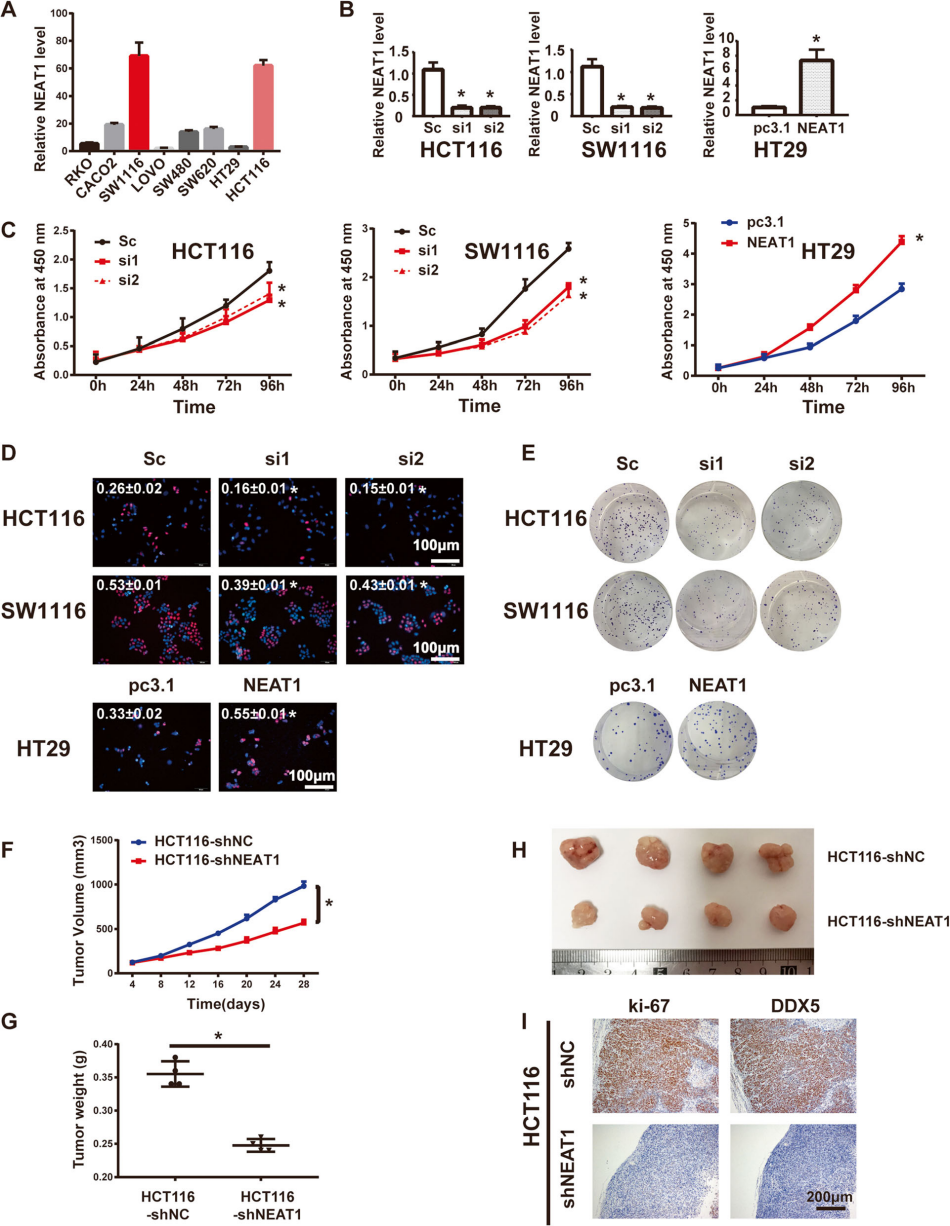
NEAT1 promoted CRC cell proliferation in vitro and in vivo. **a** The baseline NEAT1 RNA levels in eight CRC cell lines detected by RT-qPCR. **b** The efficiency of NEAT1 knockdown or overexpression was detected by RT-qPCR in the indicated cells transfected with siRNAs or plasmids (*p < 0.05). **c** CCK8 and **d** EdU assays showing that knockdown of NEAT1 suppressed cell proliferation in the HCT116 and SW1116 cell lines (*p < 0.05) and that upregulation of NEAT1 promoted cell proliferation in the HT29 cell line (*p < 0.05). **e** Colony formation assays for the indicated cells after transfection with siRNAs or plasmids (*p < 0.05). The nude mouse xenograft model showed that knockdown of NEAT1 decreased tumor growth (**f**) and the tumor weights (**g**) compared with those of the HCT116-shNC cells (*p < 0.05). **h** Representative images of tumors in nude mice. **i** Representative images of IHC staining for ki-67 and DDX5 (**Sc** represents scramble)

To assess the functional role of NEAT1 in colorectal cancer cells, first we examined the baseline NEAT1 RNA levels in eight CRC cell lines (RKO, CACO2, SW1116, LOVO, SW480, SW620, HT29, and HCT116) by RT-qPCR (Fig. 2a). NEAT1 was expressed at much higher levels in the HCT116 and SW1116 cells and relatively lower levels in the HT29 cells and was mainly located in the nucleus (Additional file 3: Figure S1A). Next, HCT116 and SW1116 cells were transfected with the NEAT1 siRNA and HT29 cells were transfected with the NEAT1 plasmid. The knockdown and overexpression efficiencies were verified by RT-PCR (Fig. 2b). The CCK8 assay showed that downregulation of NEAT1 significantly attenuated cell proliferation of the HCT116 and SW1116 cells, whereas forced NEAT1 expression had the opposite effect in the HT29 cells (Fig. 2c). This result was also confirmed by the EdU and colony formation assays (Fig. 2d, e, Additional file 3: Figure S1B-C).

Furthermore, our results showed that repression of NEAT1 induced late apoptosis (Additional file 4: Figure S2A-B) and G0/G1 phase arrest (Additional file 4: Figure S2D) in HCT116 and SW1116 cells. The western blotting analysis verified increased PARP1 and cleaved caspase 3 expression (Additional file 4: Figure S2C) and decreased cyclin D1 and p27 expression. In contrast, the G2/M transition-related markers cyclin B1 and CDC25B were not obviously altered (Additional file 4: Figure S2E). These results suggested that NEAT1 promoted CRC cell proliferation by reducing cell apoptosis and inducing the G1 to S phase cell cycle transition.

To further validate these effects in vivo, we injected HCT116-shNEAT1 and HCT116-shNC cells into the subcutis of nude mice. Consistent with our in vitro results, the volumes and weights of the tumors formed by the HCT116-shNEAT1 cells were significantly smaller than those formed by the control cells (Fig. 2f–h). The Ki-67 index was lower in the NEAT1 knockdown groups (Fig. 2i). These results suggested that NEAT1 promoted the tumorigenesis of CRC cells both in vitro and in vivo.

### Altered NEAT1 affected CRC cell migration and invasion in vitro and in vivo

**Fig. 3:**
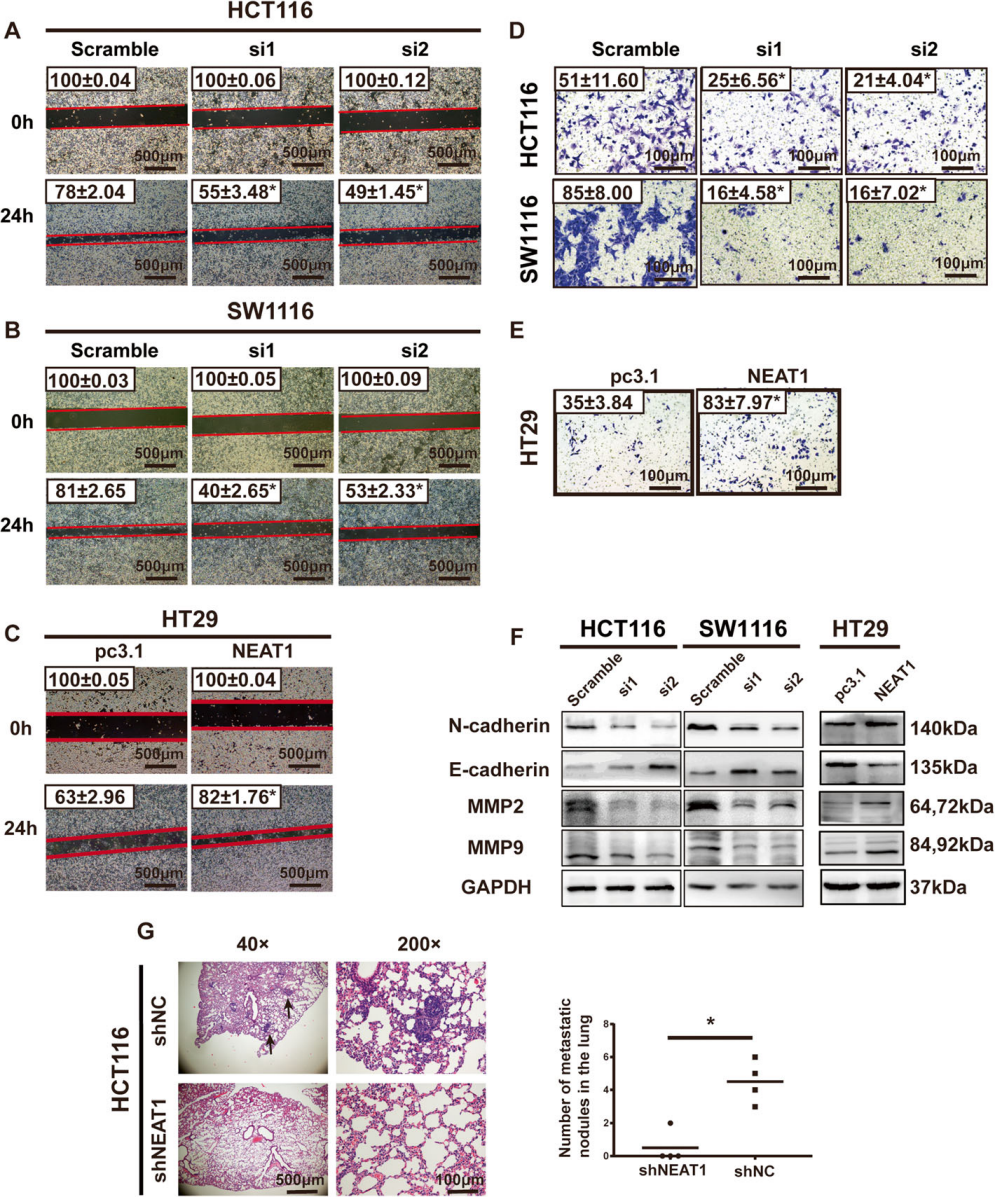
Repression of NEAT1-inhibited cell invasion and migration in vitro and in vivo. Representative images (× 40) of wound healing assays in HCT116 (**a**), SW1116 (**b**), and HT29 (**c**) cells (*p < 0.05). **d** Representative images (× 200) of Transwell invasion assays for the indicated cells (*p < 0.05). **e** Representative images of Transwell invasion assays for HT29 cells. **f** Western blotting results for N-cadherin, E-cadherin, MMP2, and MMP9. **g** Representative images of lung metastasis in nude mice with HE staining

To determine the effect of altered NEAT1 expression on the migration and invasion of CRC cells, NEAT1-siRNA-transfected and NEAT1 plasmid-transfected CRC cells were wounded by scratching and maintained for 24 h. Knockdown of NEAT1 significantly inhibited the flattening and spreading abilities of the HCT116 and SW1116 cells (Fig. 3a, b, Additional file 5: Figure S3A), whereas overexpression of NEAT1 strongly promoted the flattening and spreading abilities of the HT29 cells (Fig. 3c, Additional file 5: Figure S3A). This result was also confirmed by the Transwell assay. We found that the invasive abilities of the NEAT1-siRNA-transfected HCT116 and SW1116 cells were significantly inhibited (Fig. 3d, Additional file 5: Figure S3B), whereas the invasive ability was higher in the NEAT1-transfected HT29 cells than in the control cells (Fig. 3e, Additional file 5: Figure S3B). Accordingly, the RT-qPCR and western blotting results showed that downregulation of NEAT1 resulted in higher E-cadherin expression and lower N-cadherin expression at both the mRNA and protein levels (Fig. 3f, Additional file 5: Figure S3C). The cell migration and invasion markers MMP2 and MMP9 were also decreased when NEAT1 was downregulated (Fig. 3f). However, overexpression of NEAT1 led to increased N-cadherin, MMP2, and MMP9 and decreased E-cadherin expression (Fig. 3f).

Next, we evaluated the functional role of NEAT1 in CRC cell metastasis in vivo*.* We injected HCT116-shNEAT1 and HCT116-shNC cells into the tail veins of mice in groups of four. The number of lung metastatic tumor nodules was decreased in the HCT116-shNEAT1 group compared with that of the HCT116-shNC group (Fig. 3g). Consistent with the effects of NEAT1 expression on migration and invasion in vitro, NEAT1 knockdown significantly abrogated metastasis both in vitro and in vivo.

### NEAT1 interacted and enhanced with DDX5 stability

**Fig. 4:**
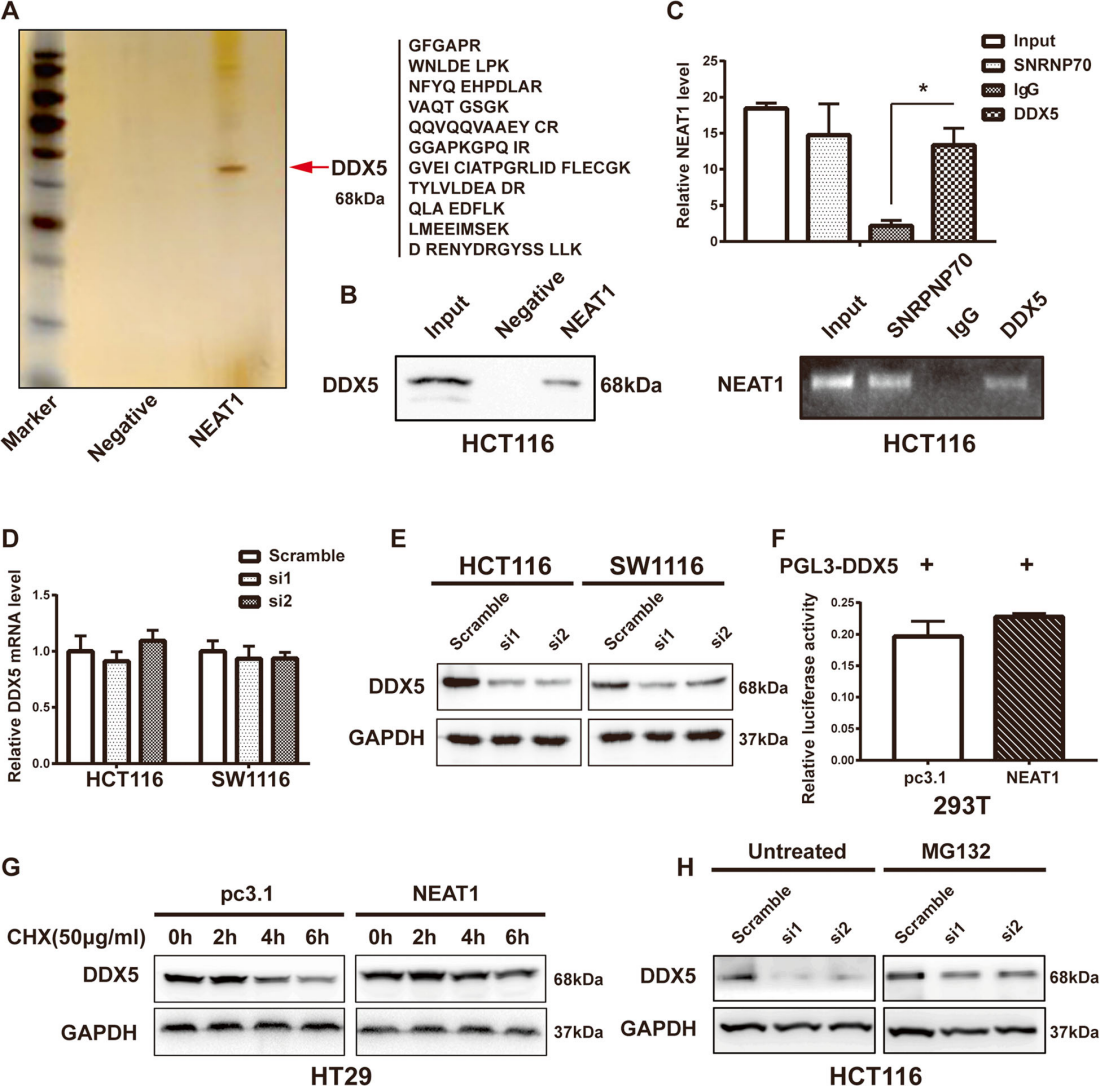
NEAT1 binds to the DDX5 protein and enhances its stability. **a** RNA pull-down assay after silver staining and **b** western blotting to detect DDX5 protein expression in HCT116 cells. **c** RIP assay showing that DDX5 interacted with NEAT1 in HCT116 cells. The RT-qPCR products were analyzed by electrophoresis (below) (*p < 0.05). **d** The DDDX5 mRNA and **e** protein levels after transfection of the cells with siRNAs (*p < 0.05). **f** Dual-Luciferase Assays to assess DDX5 promoter activity. **g** Western blotting showing the DDX5 protein level after treatment of the cells with CHX (50 μg/mL) (*p < 0.05). **h** Western blotting showing the DDX5 protein level after treatment of the cells with or without MG132 (10 μmol/mL)

As described above, NEAT1 plays an important role in CRC progression, although the detailed mechanism remains unknown. Because lncRNAs can interact with proteins, a biotin RNA-protein pull-down assay was performed to identify potential proteins binding to NEAT1 (Fig. 4a). Interestingly, DDX5 was identified as an interacting target of NEAT1 by MS analysis (Additional file 6: Table S6), and an immunoprecipitation assay further confirmed that DDX5 directly bound to NEAT1 (Fig. 4b). Intriguingly, the RIP assay confirmed the interaction between DDX5 and NEAT1 in extracts from HCT116 cells. SNRNP70 was used as the positive control (Fig. 4c). Collectively, these results demonstrated a direct interaction between DDX5 and NEAT1.

Next, we analyzed the regulatory effects of NEAT1 on DDX5. We found that downregulation of NEAT1 effectively reduced the DDX5 protein level but not the mRNA level in the HCT116 and SW1116 cells (Fig. 4d, e). In addition, immunohistochemistry analysis of xenografts showed that nude mice in the NEAT1-knockdown group exhibited lower DDX5 expression (Fig. 2i). To further clarify the mechanism underlying the regulation of DDX5 expression by NEAT1, first we examined whether NEAT1 could directly regulate the transcriptional activity of DDX5. Our results showed that DDX5 promoter activity was not increased in NEAT1-transfected HCT116 cells (Fig. 4f), suggesting that NEAT1 might participate in regulation of DDX5 at the posttranscriptional level. Therefore, we used the protein synthesis inhibitor cycloheximide (CHX) to observe the effect of NEAT1 on DDX5 degradation. The western blotting results showed that overexpression of NEAT1 in HT29 cells enhanced DDX5 protein stability (Fig. 4g). Moreover, the 26S proteasome inhibitor MG132 rescued the reduction of DDX5 caused by repression of NEAT1 in HCT116 cells (Fig. 4h), suggesting that NEAT1 elevated DDX5 by reducing its degradation. Taken together, our data indicated that NEAT1 directly bound the DDX5 protein and enhanced its stability in CRC cells.

### NEAT1 activated Wnt/β-catenin signaling by targeting DDX5

**Fig. 5:**
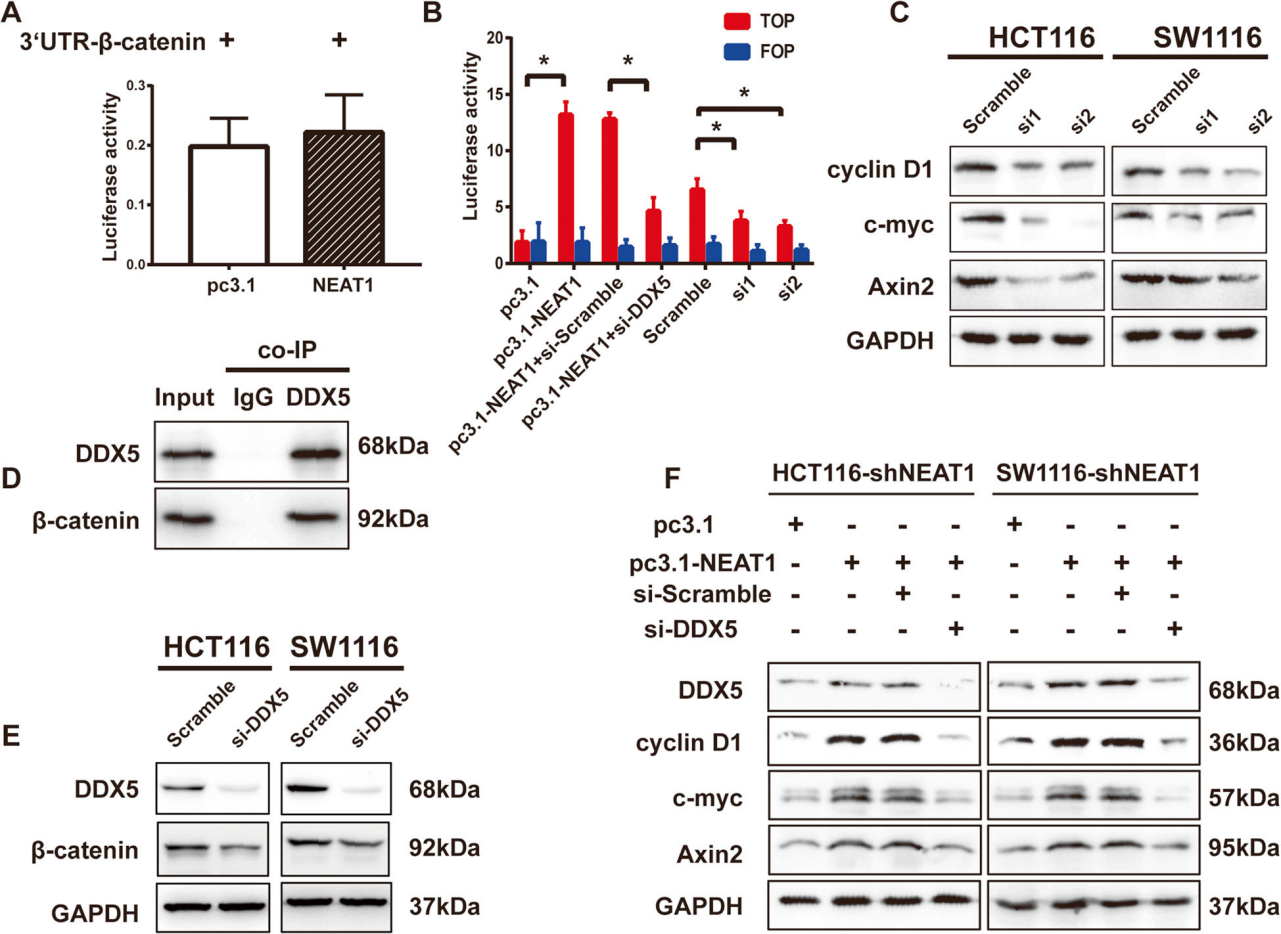
The influence of NEAT1 on β-catenin activation was dependent on DDX5. Dual-Luciferase Assays for β-catenin 3′-UTR (**a**) and TOP/FOP activity (**b**). **c** The Axin2, cyclin D1, and c-myc protein levels in the indicated cells (*p < 0.05). **d** Co-IP to detect the interaction of endogenous DDX5 and β-catenin in HCT116 cells. **e** Knockdown of DDX5 reduced β-catenin expression. **f** The DDX5, Axin2, cyclin D1, and c-myc protein levels after rescue of NEAT1 expression in shNEAT1 stable cells with or without si-DDX5

Recently, decreased NEAT1 expression was reported to inhibit Wnt/β-catenin signaling pathway activity in glioblastoma [21]. In our study, we performed a TOP/FOP-flash luciferase assay; the results revealed that Wnt/β-catenin signaling was inhibited by NEAT1 depletion (Fig. 5b). However, the Dual-Luciferase Reporter Assay showed that NEAT1 did not influence the activity of the β-catenin 3′-UTR (Fig. 5a), which indicated that NEAT1 did not regulate β-catenin directly. DDX5 reportedly forms a complex with β-catenin and promotes its transcriptional ability to activate gene transcription [22]. Co-IP assays detected an interaction between endogenous DDX5 and β-catenin (Fig. 5d) in HCT116 cells, and knockdown of DDX5 reduced the β-catenin level (Fig. 5e). We speculated that NEAT1 might activate Wnt/β-catenin signaling by targeting DDX5. To explore the effects of NEAT1 on Wnt/β-catenin signaling, we examined the levels of the Wnt/β-catenin signaling targets Axin2, c-myc, and cyclin D1. The RT-qPCR and western blotting analyses showed that downregulation of NEAT1 reduced the mRNA (Additional file 5: Figure S3D) and protein (Fig. 5c) levels of Wnt/β-catenin signaling target genes. Furthermore, the increased TOP/FOP-flash luciferase activity resulting from NEAT1 overexpression was significantly suppressed by si-DDX5 (Fig. 5b), suggesting that NEAT1-induced activation of Wnt/β-catenin signaling was dependent on DDX5 expression. The RT-qPCR and western blotting analyses showed that downregulation of DDX5 did not reduce the NEAT1 RNA level but did abrogate the increase in the Axin2, c-myc, and cyclin D1 levels (Fig. 5f, Additional file 5: Figure S3E). Collectively, our results suggest that NEAT1 indirectly promotes β-catenin transcriptional activation by binding to DDX5.

### NEAT1 facilitated tumor proliferation and metastasis in a DDX5-mediated manner

**Fig. 6:**
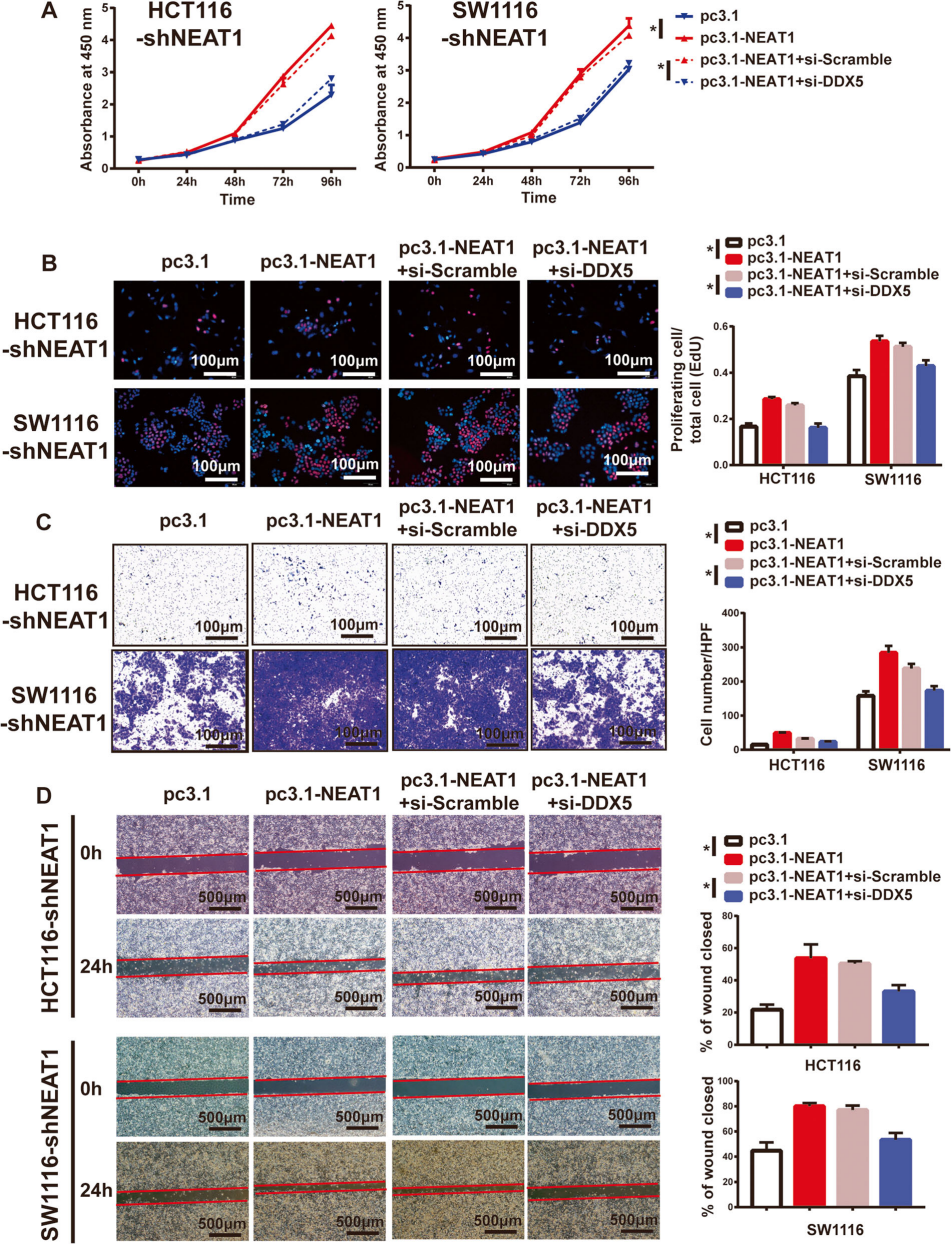
NEAT1 facilitated CRC cell progression in a DDX5-mediated manner. **a** CCK-8 and **b** EdU assay results showing that knockdown of DDX5 partially attenuated the enhanced cell proliferation induced by overexpression of NEAT1 in HCT116-shNEAT1 and SW1116-shNEAT1 cells (*p < 0.05). Representative images of the **c** Transwell invasion assays and **d** wound healing assays showing that DDX5 repression rescued the enhanced invasion and migration abilities induced by NEAT1 overexpression (*p < 0.05)

To elucidate whether NEAT1 functioned in CRC cells in a DDX5-mediated manner, we performed CCK-8 and EdU assays. The results showed that overexpression of NEAT1 recovered the proliferation potential of HCT116 and SW1116 NEAT1 stable knockdown cells, which nevertheless was impaired by the simultaneous downregulation of DDX5 (Fig. 6d, e). Similarly, the effect of NEAT1 on the invasion and migration of CRC cells was also partially attenuated by repression of DDX5 (Fig. 6c, d). Therefore, we hypothesized that NEAT1 facilitated tumor proliferation and metastasis in a DDX5-mediated manner.

### Clinical associations between NEAT1 and DDX5 in human CRC samples

**Fig. 7:**
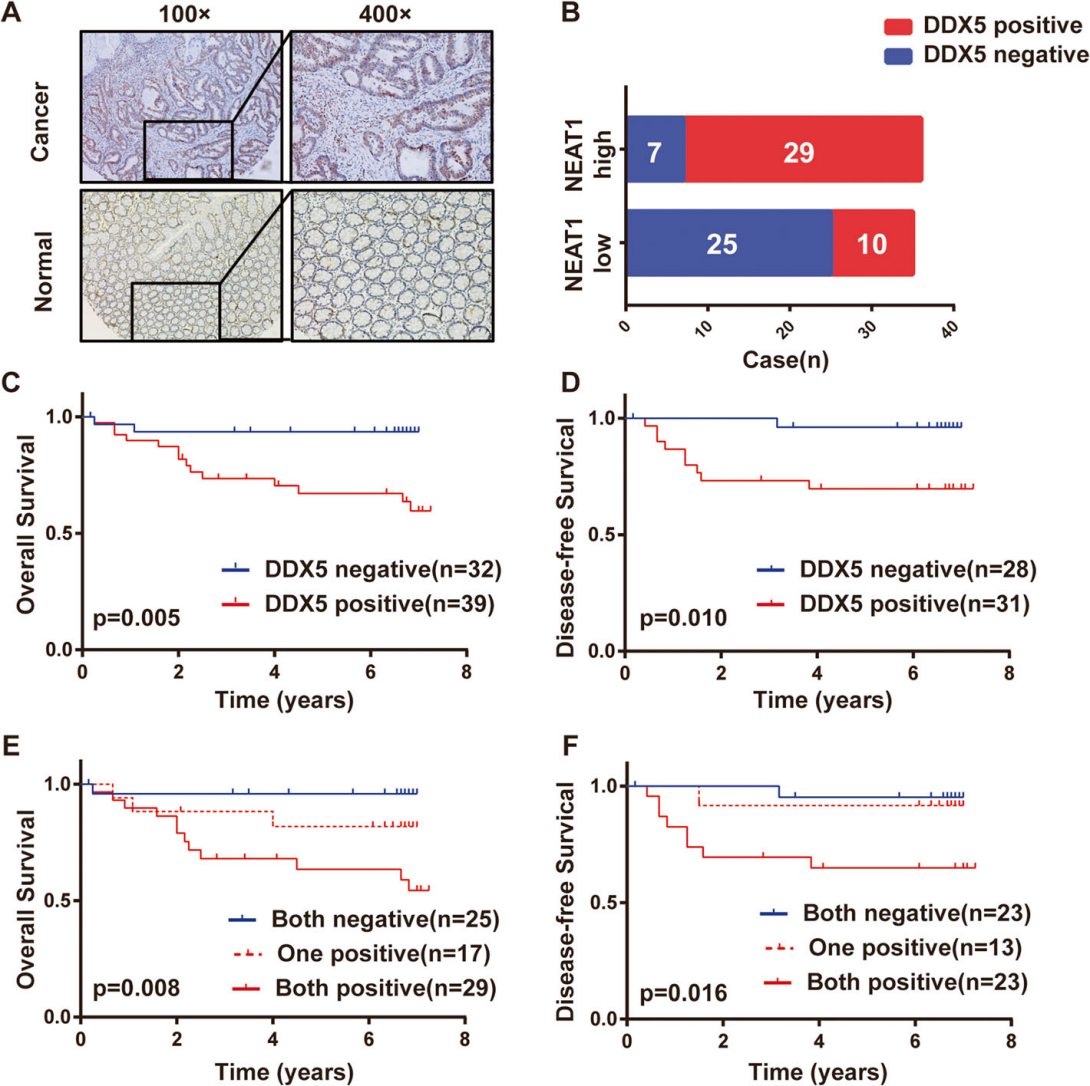
NEAT1 and DDX5 expression in clinical CRC samples. **a** Representative images of DDX5 detected by IHC. **b** The chi-square test identified an association between DDX5 and NEAT1 in the CRC samples (*n* = 71, *p* < 0.01). **c**–**f** Kaplan-Meier analyses of the FDUSCC dataset. Patients with high DDX5 expression had poorer OS (**c**) and DFS (**d**) than those with negative expression. The patients were divided into three groups based on NEAT1 and DDX5 expression (negative or positive). Both positive groups had the poorest prognoses with the lowest OS (**e**) and DFS (**f**)

Finally, we tested the clinical association between NEAT1 and DDX5 in the FDUSCC dataset. Immunohistochemistry analysis of CRC samples showed that DDX5 was located in the cell nucleus and was overexpressed in cancerous tissues compared to normal tissues (Fig. 7a). In addition, DDX5 expression was correlated with NEAT1 expression in 71 CRC samples (*p* < 0.01, Fig. 7b). The survival analysis demonstrated that CRC patients with positive DDX5 expression (H score ≥ 50) had poorer OS and DFS than those with negative DDX5 expression (Fig. 7c, d). Next, the patients were divided into three groups based on their NEAT1 and DDX5 expression levels. Patients with positive NEAT1 and DDX5 expression had the poorest OS and DFS. In contrast, those with negative NEAT1 and DDX5 expression had the best OS (*p* = 0.008) and DFS (*p* = 0.016) (Fig. 7e, f). In addition, the multivariate COX analysis showed that the combination of NEAT1 and DDX5 was an independent prognostic indicator of OS (*p* = 0.024, HR = 6.916, 95% CI 1.291–37.051, Additional file 2: Table S5).

## Discussion

Nuclear lncRNAs participate in many critical biological processes and are often dysregulated in a variety of cancers, including CRC. However, further investigations are required to elucidate how individual lncRNAs function [23]. Our study contributes to understanding the role of NEAT1 upregulation in CRC progression. Our results suggest that NEAT1 promotes CRC tumor growth and metastasis by stabilizing the DDX5 protein, thereby activating β-catenin gene transcription.

The role of NEAT1 in tumors seems to be controversial. Some studies have shown that NEAT1 is an oncogene in various cancers, such as lung cancer, breast cancer, prostate cancer, colorectal cancer, and pancreatic cancer [12, 24–26]. In prostate cancer, *Chakravarty D* et al. [24] demonstrated that NEAT1 was recruited to the promoters of well-characterized prostate cancer-related genes and contributed to an epigenetic “on” state. Other studies have shown that NEAT1 acts as a tumor suppressor and a target of p53. *Adriaens* et al. [27] showed that NEAT1 promoted ATR signaling in response to replication stress and was engaged in a negative feedback loop that attenuated oncogene-dependent activation of p53. *Blume CJ* et al. [28] indicated that NEAT1 and lincRNA-p21 were induced in response to DNA damage in the presence of functional p53 but not in chronic lymphocytic leukemia with a p53 mutation. *Masashi Idogawa* et al. [29] showed that p53 could induce NEAT1 expression in cells with wild-type p53, such as A549 and MCF7, but that NEAT1 was not increased at all in HCT116 (p53−/−) cells. Therefore, the role of NEAT1 in tumor cells may be cell-type dependent, although this possibility needs to be further studied.

NEAT1 is an essential component of nuclear paraspeckles [30], which contain ribonucleoprotein complexes formed around NEAT1 [9]. At present, NEAT1 is thought to be exclusive to the nucleus and mainly function as a transcriptional regulator. In our study, we found that NEAT1 was mainly located in the nucleus with a small amount in the cytoplasm, which was consistent with the report of Chiu et al. [31]. Furthermore, we found that NEAT1 interacted with DDX5 and enhanced its stability posttranslationally, which seemed to be inconsistent with its traditional function; however, a number of posttranscriptional functions for nuclear lncRNAs are also emerging (e.g., HOTAIR assists in assembling the chromatin modification complex in the nucleus). Yoon JH et al. [32] showed evidence that HOTAIR promoted the ubiquitination of Ataxin-1 by Dzip3 and Snurportin-1 by Mex3b and increased their degradation. Because Dzip3 was localized in the cytoplasm, the enhanced ubiquitination of Ataxin-1 might be linked to the cytoplasmic presence of HOTAIR. Mex3b and Snurportin-1 localized in both the nucleus and the cytoplasm, which suggested that HOTAIR-facilitated ubiquitination could occur in both cellular compartments [32].

Studies have reported that acetylation and sumoylation of DDX5 increase its stability. Thus, NEAT1 may form complexes with a histone acetyltransferase or SUMO-conjugating enzymes and interact with their substrate DDX5. By facilitating formation of the complexes, NEAT1 mediates the proteolysis of DDX5.

DDX5 is a prototypical member of the DEAD box family of RNA helicases and plays important roles in multiple biological processes, including cell proliferation, early organ development, and maturation [33–35]. DDX5 exhibited clear cell cycle-related localization in the nucleus, and its expression was related to tumor progression and transformation [36]. DDX5 was determined to be overexpressed in colon cancer, and the degree of its expression was associated with progression of the disease from polyp to adenoma to adenocarcinoma [22]. DDX5 may affect β-catenin in two ways: in the cytoplasm by protecting β-catenin from degradation via dissociation from the cytoplasmic APC/axin/GSK-3β complex or in the nucleus by augmenting β-catenin transcriptional activity [22]. The IHC analyses of DDX5 expression in nude mice and the tissue microarrays in our study indicated that the latter possibility was more likely. DDX5 can directly bind β-catenin and TCF4 to activate β-catenin transcription [22, 37, 38]. To the best of our knowledge, β-catenin plays important roles in CRC progression. Thus, we investigated whether NEAT1 affected β-catenin in a manner that was dependent on or independent of DDX5. Because NEAT1 was located in the cell nucleus, we conceived that it might affect β-catenin transcriptional activity. Our results confirmed that NEAT1 indirectly promoted β-catenin transcriptional activation in a manner that was dependent upon DDX5. Furthermore, NEAT1 regulated DDX5 expression by enhancing its protein stability.

Finally, our study investigated the clinical significance of NEAT1 and DDX5. NEAT1 was positively correlated with DDX5 expression in 71 CRC patients. Interestingly, although NEAT1 alone could not predict the DFS of CRC patients, patients with high NEAT1 expression together with positive DDX5 expression had the worst OS and DFS. These findings highlight that NEAT1 acts as an onco-lncRNA by regulating DDX5 in CRC.

## Conclusions

In summary, our study demonstrated important roles of NEAT1 in CRC progression and showed that NEAT1 activated β-catenin transcriptional activity by directly binding DDX5, which might reflect the underlying molecular mechanisms of their biological functions. These findings provide new insights into the roles of lncRNAs in CRC progression. Together with further research, these findings may prove to be clinically useful strategies for CRC treatment.

## Additional files

Additional file 1: Supplemental materials and methods. (DOCX 20 kb)

Additional file 2: **Table S1.** Primary antibodies for western blot. **Table S2.** Primers for real-time PCR. **Table S3.** Association between clinicopathological features and NEAT1 expression. **Table S4.** Univariate Cox proportional hazards model for overall survival (OS) and disease-free survival (DFS). **Table S5.** Multivariate Cox proportional hazards model for OS and DFS. (DOCX 29 kb)

Additional file 3: **Figure S1.** (A) NEAT1 was mainly located in nuclear. (B) Column chart of EdU assay of indicated CRC cells (*p < 0.05). (C) Quantitative results for colony formation assay of indicated CRC cells (*p < 0.05). (TIF 16126 kb)

Additional file 4: **Figure S2.** (A–B). Knockdown of NEAT1 increased the proportion of late apoptotic cells (*p < 0.05). (C) Western blot showed that knockdown of NEAT1 enhanced PARP1 and caspase-3 cleavage. (D) Repression of NEAT1 inhibited the transition from the G0/G1 to the S phase of the cell cycle (*p < 0.05). (E) Western blot showed the reduction of cyclin D1 and p27 after NEAT1 repression. (c-PARP1 represents cleaved-PARP1; c-caspase3 represents cleaved-caspase3). (TIF 1695 kb)

Additional file 5: **Figure S3.** Quantitative results for wound healing assays (A) and Transwell assays (B) of indicated CRC cells. (C) The mRNA level of N-cadherin and E-cadherin (*p < 0.05). (D) The mRNA level of Axin2, cyclin D1, and c-myc for indicated cells (*p < 0.05). (E) The mRNA level changes of DDX5, Axin2, cyclin D1, and c-myc after NEAT1 expression rescued in shNEAT1 stable cells with or without si-DDX5(*p < 0.05). (Sc represents scramble) (TIF 986 kb)

Additional file 6: **Table S6.** Mass spectrometry analysis for RNA pull-down. (XLSX 78 kb)

## Abbreviations

CHX: Cycloheximide
CRC: Colorectal cancer
DFS: Disease-free survival
FDUSCC: Fudan University Shanghai Cancer Center
MS: Mass spectrometry
OS: Overall survival
RIP: RNA immunoprecipitation
