# Supplementary material for: The lncRNA NEAT1 activates Wnt/β-catenin signaling and promotes colorectal cancer progression via interacting with DDX5
Source: J Hematol Oncol. 2018 Sep 5;11:113. doi: 10.1186/s13045-018-0656-7 (PMC6125951; doi:10.1186/s13045-018-0656-7)
Supplement: Supplementary file 1 — Supplemental materials and methods. (DOCX 20 kb) [file 13045_2018_656_MOESM1_ESM.docx]

**Supplemental materials and methods**

**Cell lines**

A series of human CRC cell lines (HT29, RKO, SW480, SW620, LoVo, HCT116, SW1116, Caco2) were purchased from the Cell Bank of Shanghai Institute of Biochemistry and Cell Biology (Shanghai, China). The cell lines were cultured in DMEM (HyClone, USA) supplement with 10% FBS (Gibco, USA) and 1 % complex of penicillin and streptomycin in a 5% CO_2_ incubator at 37 ℃.

**RNA extraction, reverse transcription (RT) and real-time qPCR**

Total RNA was extracted by Trizol ((Invitrogen, CA, USA) according to the manufacture’s instruction. Then 500 ng RNA was transcript into cDNA using the PrimeScript RT reagent kit (Takara, Shiga, Japan). Quantitative real-time PCR was completed with SYBR Premix Ex Taq II (Takara, Shiga, Japan) according to manufacturer’s protocol. GAPDH was used as the internal control to normalize the mRNA level. Relative mRNA level was calculated by 2^-ΔΔCt^ comparative method. Related primer sequences was summarized in Supplementary Table 2.

**Proliferation and colony formation assay**

Cells transfected with vectors or siRNAs were re-suspended and seeded into 96-well plates in a density of 5000 cells/well in triplicate. CCK-8 reagent (10 μl, Dojindo, Japan) was added to each well at 0, 24, 48, 72 and 96 h. The absorbance at 450 nm was measured after 2 h incubation at 37 ℃. EdU DNA assay was performed using EdU DNA imaging kit (Invitrogen, CA, USA) according to the protocol. Microscopic images of cells were observed under Leica inverted fluorescence microscope (Leica, Microsystems, Wetzlar, Germany), and photographed by ProgRes Image Capture Software (JENOPTIK Optical Systems, Germany). Blue color represents the nuclei and red color indicate EdU positive nuclei, and the results were quantified by counting ten random fields.

For the colony formation assay, single-cell suspension solutions were prepared and seeded into 6-well plates with a density of 500/well and cultured in complete medium for 14 days. Developed cell colonies were fixed, stained and then photographed.

**Apoptosis and cell cycle assays**

For cell apoptosis and cell cycle analysis, cells were collected 48 h after transfection. PE Annexin V Apoptosis Detection Kit I (BD Biosciences, NJ, USA) was used to assess cell apoptosis. Simply, PE Annexin-V and 7-AAD was used to label early and late apoptotic/death cells, respectively. For cell cycle analysis, cells were fixed with ethanol at -20 ℃ overnight, then washed and stained with PI. Treated Cells for apoptosis and cell cycle analysis were analyzed by Beckman Cytomics FC 500 (Beckman Coulter, CA, USA).

**Cell migration and invasion assays**

The cell migration ability was accessed by wound-healing assay. Linear wound was generated with a 200 μl pipette tip until cell confluence. Wound closure was examined and photographed at pre-determined time points (0, 24 h) in multiple microscopic regions.

And the cell invasion ability was accessed by the Transwell assay using 24-Well Cell Invasion Assay (Corning, NY, USA) with Matrigel (BD Biosciences, NJ, USA). 24 h after transfection, 3×10^4^ cells with FBS-free medium were seeded into the upper chamber, and medium containing 20% FBS was applied to the lower chamber as chemoattractant. After 48 h incubation, cells attached to the lower surface of the chamber were fixed with ethanol, stained with 0.5% crystal violet, and then photographed.

**Supplementary Figure Legends**

**Figure S1.** **(A)** NEAT1 was mainly located in nuclear. **(B)** Column chart of EdU assay of indicated CRC cells (* p<0.05). **(C)** Quantitative results for colony formation assay of indicated CRC cells (* p<0.05).

**Figure S2. (A-B)** Knockdown of NEAT1 increased the proportion of late apoptotic cells (* p<0.05). **(C)** Western blot showed that knockdown of NEAT1 enhanced PARP1 and caspase-3 cleavage. **(D)** Repression of NEAT1 inhibited the transition from the G0/G1 to the S phase of the cell cycle (* p<0.05). **(E)** Western blot showed the reduction of cyclin D1 and p27 after NEAT1 repression. (c-PARP1 represents cleaved-PARP1; c-caspase3 represents cleaved-caspase3)

**Figure S3.** Quantitative results for wound healing assays **(A)** and transwell assays **(B)** of indicated CRC cells. **(C)** The mRNA level of N-cadherin and E-cadherin (* p<0.05). **(D)** The mRNA level of Axin2, cyclin D1, and c-myc for indicated cells (* p<0.05). **(E)**The mRNA level changes of DDX5, Axin2, cyclin D1, and c-myc after NEAT1 expression rescued in shNEAT1 stable cells with or without si-DDX5(* p<0.05). (Sc represents scramble)
