# Supplementary material for: The lncRNA NEAT1 activates Wnt/β-catenin signaling and promotes colorectal cancer progression via interacting with DDX5
Source: J Hematol Oncol. 2018 Sep 5;11:113. doi: 10.1186/s13045-018-0656-7 (PMC6125951; doi:10.1186/s13045-018-0656-7)
Supplement: Supplementary file 2 — Table S1. Primary antibodies for western blot. Table S2. Primers for real-time PCR. Table S3. Association between clinicopathological features and NEAT1 expression. Table S4. Univariate Cox proportional hazards model for overall survival (OS) and disease-free survival (DFS). Table S5. Multivariate Cox proportional hazards model for OS and DFS. (DOCX 29 kb) [file 13045_2018_656_MOESM2_ESM.docx]

**Table S1** Primary antibodies for Western blot.

| Antibody | Concentration | Specificity | Company |
| --- | --- | --- | --- |
| DDX5 | 1:1000 | Rabbit | Cell Signaling Technology |
| β-catenin | 1:1000 | Rabbit | Cell Signaling Technology |
| cyclin D1 | 1:1000 | Rabbit | Cell Signaling Technology |
| c-myc | 1:1000 | Rabbit | Cell Signaling Technology |
| Axin2 | 1:1000 | Rabbit | Cell Signaling Technology |
| E-cadherin | 1:1000 | Rabbit | Cell Signaling Technology |
| N-cadherin | 1:1000 | Rabbit | Cell Signaling Technology |
| MMP2 | 1:1000 | Rabbit | Cell Signaling Technology |
| MMP9 | 1:1000 | Rabbit | Cell Signaling Technology |
| Caspase3 | 1:1000 | Rabbit | Cell Signaling Technology |
| Cleved-caspase3 | 1:1000 | Rabbit | Cell Signaling Technology |
| PARP1 | 1:1000 | Rabbit | Cell Signaling Technology |
| Cleaved-PARP1 | 1:1000 | Rabbit | Cell Signaling Technology |
| cyclin B1 | 1:1000 | Rabbit | Cell Signaling Technology |
| p27 | 1:1000 | Rabbit | Cell Signaling Technology |
| CDC25B | 1:1000 | Rabbit | Cell Signaling Technology |
| GAPDH | 1:10000 | Rabbit | Epitomics |

**Table S2** Primers for real-time PCR.

| Primer name | Forward (5’-3’) | Reverse (5’-3’) |
| --- | --- | --- |
| NEAT1 | CCAGTTTTCCGAGAACCAAA | ATGCTGATCTGCTGCGTATG |
| DDX5 | TGAGCGACCTTATCTCTGTGC | GGTCATCCTTCATGCCTCCT |
| c-myc | GGACTATCCTGCTGCCAAGA | CGCCTCTTGACATTCTCCTC |
| cyclin D1 | GTGCTGCGAAGTGGAAACC | ATCCAGGTGGCGACGATCT |
| Axin2 | CTGGGGGCAGCGAGTATTAC | GCCTTTCCCATTGCGTTTGG |
| GAPDH | AGCCACATCGCTCAGACAC | GCCCAATACGACCAAATCC |

**Table S3** Association between clinicopathological features and NEAT1 expression.

| **Parameters** | **n** | **NEAT1 RNA level** | | ***P* Value** | |
| --- | --- | --- | --- | --- | --- |
|  |  | **Low** | **High** | |  |
| **Cases (n, %)** | 71 | 35(49.3) | 36(50.7) | |  |
| **Age, years (n, %)** |  |  |  | |  |
| <59 | 35 | 18(51.4) | 17(48.6) | | 0.723 |
| ≥59 | 36 | 17(47.2) | 19(52.8) | |  |
| **Gender (n, %)** |  |  |  | |  |
| Male | 40 | 18(45.0) | 22(55.0) | | 0.411 |
| Female | 31 | 17(54.8) | 14(45.2) | |  |
| **T stage (n, %)** |  |  |  | |  |
| T1-T3 | 15 | 8(53.3) | 7(46.7) | | 0.725 |
| T4 | 56 | 27(48.2) | 29(51.8) | |  |
| **N stage (n, %)** |  |  |  | |  |
| N0 | 27 | 14(51.9) | 13(48.1) | | 0.745 |
| N1 | 27 | 14(51.9) | 13(48.1) | |  |
| N2 | 17 | 7(41.2) | 10(58.8) | |  |
| **M stage (n, %)** |  |  |  | |  |
| M0 | 58 | 30(51.7) | 28(48.3) | | 0.387 |
| M1 | 13 | 5 (38.5) | 8(61.5) | |  |
| **AJCC stage (n, %)** |  |  |  | |  |
| Ⅰ+Ⅱ | 23 | 13(56.5) | 10(43.5) | | 0.399 |
| Ⅲ+Ⅳ | 58 | 22(45.8) | 26(54.2) | |  |
| **Differentiation (n, %)** |  |  |  | |  |
| High | 5 | 2(40.0) | 3(60.0) | | 0.310 |
| Moderate | 51 | 28(54.9) | 23(45.1) | |  |
| Low | 15 | 5(33.3) | 10(66.7) | |  |
| **Vascular invasion (n, %)** |  |  |  | |  |
| Yes | 22 | 13(59.1) | 9(40.9) | | 0.269 |
| No | 49 | 22(44.9) | 27(55.1) | |  |
| **Ki67 index (n, %)** |  |  |  | |  |
| Negative | 30 | 15(50.0) | 15(50.0) | | 0.919 |
| Positive | 41 | 20(48.8) | 21(51.2) | |  |

**Table S4** Univariate Cox proportional hazards model for overall survival (OS) and disease-free survival (DFS).

|  | | **OS** | | |  | **DFS** | | | |
| --- | --- | --- | --- | --- | --- | --- | --- | --- | --- |
| **Variable** | | **HR** | **95%CI** | **p Value** |  | **HR** | **95%CI** | **p Value** | |
| **Age, years** | |  |  |  |  |  |  | |  |
| <59 | —— | |  |  |  | —— |  | |  |
| ≥59 | 1.049 | | 0.404-2.723 | 0.921 |  | 1.680 | 0.491-5.744 | | 0.408 |
| **Gender** | |  |  |  |  |  |  | |  |
| Male | | —— |  |  |  | —— |  | |  |
| Female | | 1.539 | 0.569-4.164 | 0.396 |  | 1.017 | 0.310-3.333 | | 0.978 |
| **T stage** | |  |  |  |  |  |  | |  |
| T1-T2 | | —— |  |  |  | —— |  | |  |
| T3-T4 | | 0.858 | 0.121-6.090 | 0.878 |  | 1.014 | 0.421-2.446 | | 0.975 |
| **N stage** | |  |  |  |  |  |  | |  |
| N0 | | —— |  |  |  | —— |  | |  |
| N1 | | 2.809 | 0.725-10.875 | 0.135 |  | 0.323 | 0.072-1.448 | | 0.140 |
| N2 | | 5.308 | 1.297-19.571 | 0.020* |  | 0.624 | 0.156-2.499 | | 0.506 |
| **M stage** | |  |  |  |  |  |  | |  |
| M0 | | —— |  |  |  | NA |  | |  |
| M1 | | 11.216 | 4.116-30.567 | <0.01* |  | NA |  | |  |
| **AJCC stage** | |  |  |  |  |  |  | |  |
| Ⅰ+Ⅱ | | —— |  |  |  | —— |  | |  |
| Ⅲ+Ⅳ | | 4.697 | 2.119-10.412 | <0.01* |  | 1.554 | 0.514-4.698 | | 0.435 |
| **Differentiation** | |  |  |  |  |  |  | |  |
| High | | —— |  |  |  | —— |  | |  |
| Moderate | | 0.382 | 0.083-1.748 | 0.215 |  | 1.147 | 0.323-4.068 | | 0.832 |
| Low | | 0.708 | 0.137-3.661 | 0.680 |  | 2.350 | 0.301-18.367 | | 0.415 |
| **Vascular invasion** | |  |  |  |  |  |  | |  |
| Yes | | 0.608 | 0.198-1.866 | 0.384 |  | 0.391 | 0.084-1.812 | | 0.230 |
| No | | —— |  |  |  | —— |  | |  |
| **Ki-67 index** | |  |  |  |  |  |  | |  |
| Negative | | —— |  |  |  | —— |  | |  |
| Positive | | 0.517 | 0.197-1.359 | 0.181 |  | 0.600 | 0.183-1.967 | | 0.399 |
| **NEAT1 level** | |  |  |  |  |  |  | |  |
| High | | 4.457 | 1.267-15.685 | 0.011* |  | 4.643 | 0.985-21.887 | | 0.052 |
| Low | | —— |  |  |  | —— |  | |  |
| **DDX5 expression** | |  |  |  |  |  |  | |  |
| Negative | | —— |  |  |  | —— |  | |  |
| Positive | | 6.281 | 1.424-27.705 | 0.015* |  | 9.184 | 1.162-72.575 | | 0.036* |
| **Combination of NEAT1 and DDX5** | |  |  |  |  |  |  | |  |
| Both negative | | —— |  |  |  | —— |  | |  |
| Only NEAT1 high | | 3.477 | 0.217-55.625 | 0.378 |  | 0.000 | 0.000- | | 0.989 |
| Only DDX5 positive | | 5.252 | 0.176-57.956 | 0.176 |  | 3.121 | 0.195-49.908 | | 0.421 |
| Both positive | | 11.426 | 1.481-88.127 | 0.019* |  | 8.949 | 1.117-71.670 | | 0.039* |

*p<0.05 indicated a significance.

**Table S5** Multivariate Cox proportional hazards model for OS and DFS.

|  | | **OS** | | |  | **DFS** | | | |
| --- | --- | --- | --- | --- | --- | --- | --- | --- | --- |
| **Variable** | | **HR** | **95%CI** | **p Value** |  | **HR** | **95%CI** | **p Value** | |
| NEAT1 level | | 0.313 | 0.031-3.205 | 0.328 |  | 0.173 | 0.005-5.835 | | 0.329 |
| Combination of NEAT1 and DDX5 | 6.916 | | 1.291-37.051 | 0.024* |  | 6.665 | 0.809-54.922 | | 0.078 |
| T stage | 0.687 | | 0.263-1.791 | 0.443 |  | 1.361 | 0.302-6.126 | | 0.688 |
| N stage | | 5.700 | 1.585-20.490 | 0.008* |  | 7.195 | 1.372-37.473 | | 0.017* |
| AJCC stage | | 4.557 | 1.837-11.305 | 0.001* |  | 0.475 | 0.093-2.437 | | 0.372 |
| Ki-67 index | | 0.332 | 0.090-1.227 | 0.098 |  | 0.751 | 0.099-1.495 | | 0.385 |

*p<0.05 indicated a significance.
