# Supplementary figures and images for: The lncRNA NEAT1 activates Wnt/β-catenin signaling and promotes colorectal cancer progression via interacting with DDX5
Source: J Hematol Oncol. 2018 Sep 5;11:113. doi: 10.1186/s13045-018-0656-7 (PMC6125951; doi:10.1186/s13045-018-0656-7)

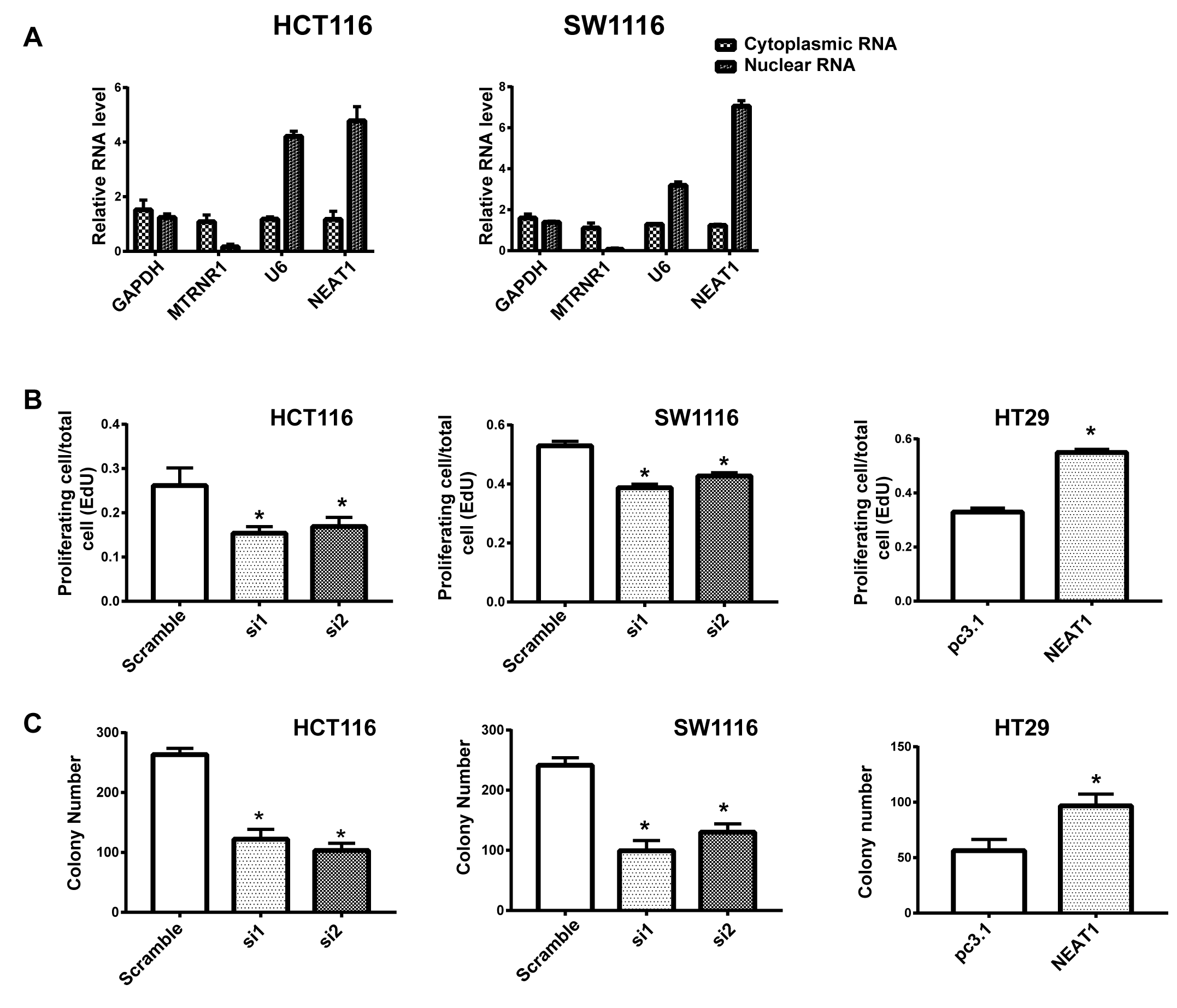

Supplement: Supplementary file 3 — Figure S1. (A) NEAT1 was mainly located in nuclear. (B) Column chart of EdU assay of indicated CRC cells (*p < 0.05). (C) Quantitative results for colony formation assay of indicated CRC cells (*p < 0.05). (TIF 16126 kb) [file 13045_2018_656_MOESM3_ESM.tif]

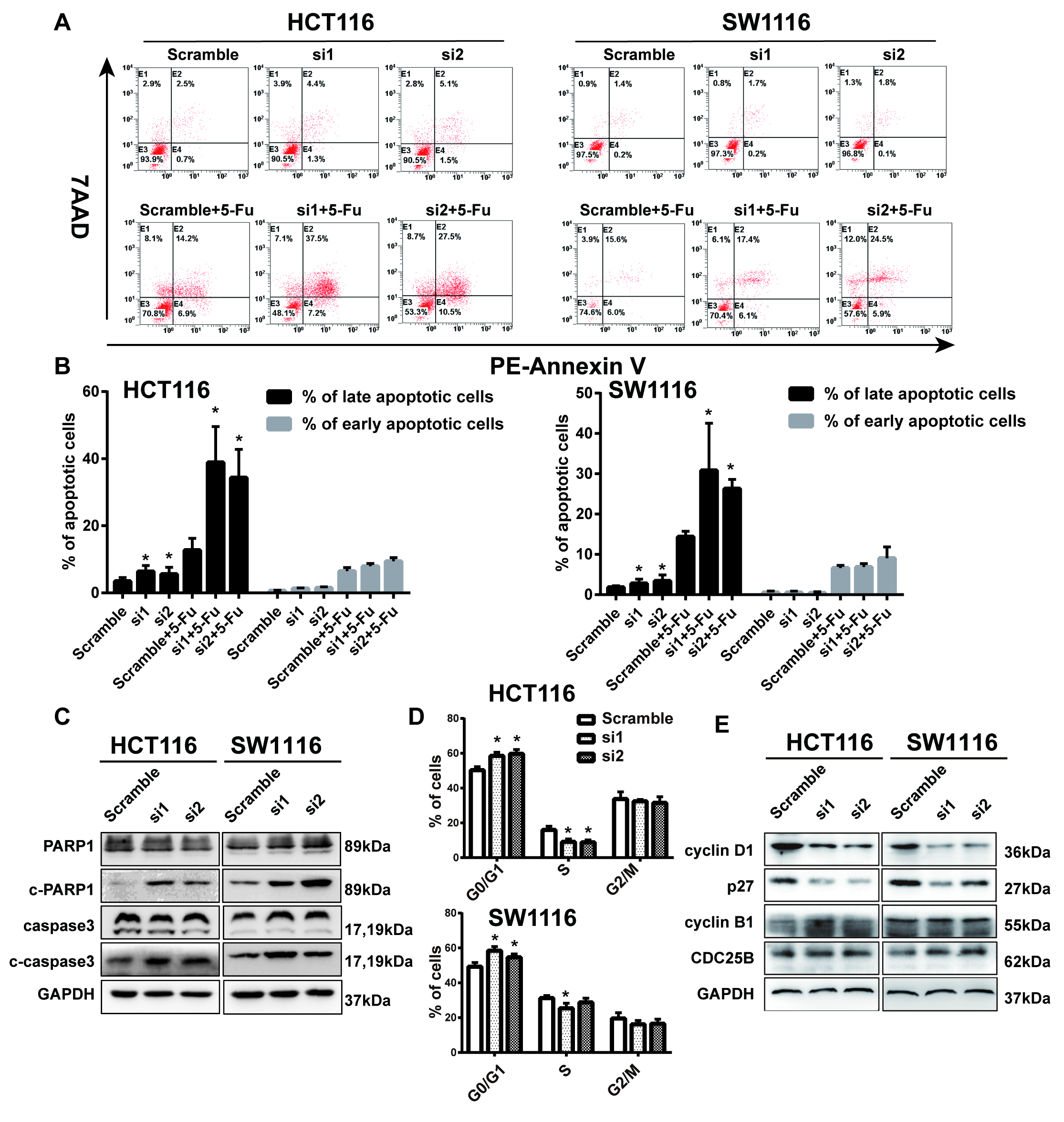

Supplement: Supplementary file 4 — Figure S2. (A–B). Knockdown of NEAT1 increased the proportion of late apoptotic cells (*p < 0.05). (C) Western blot showed that knockdown of NEAT1 enhanced PARP1 and caspase-3 cleavage. (D) Repression of NEAT1 inhibited the transition from the G0/G1 to the S phase of the cell cycle (*p < 0.05). (E) Western blot showed the reduction of cyclin D1 and p27 after NEAT1 repression. (c-PARP1 represents cleaved-PARP1; c-caspase3 represents cleaved-caspase3). (TIF 1695 kb) [file 13045_2018_656_MOESM4_ESM.tif]

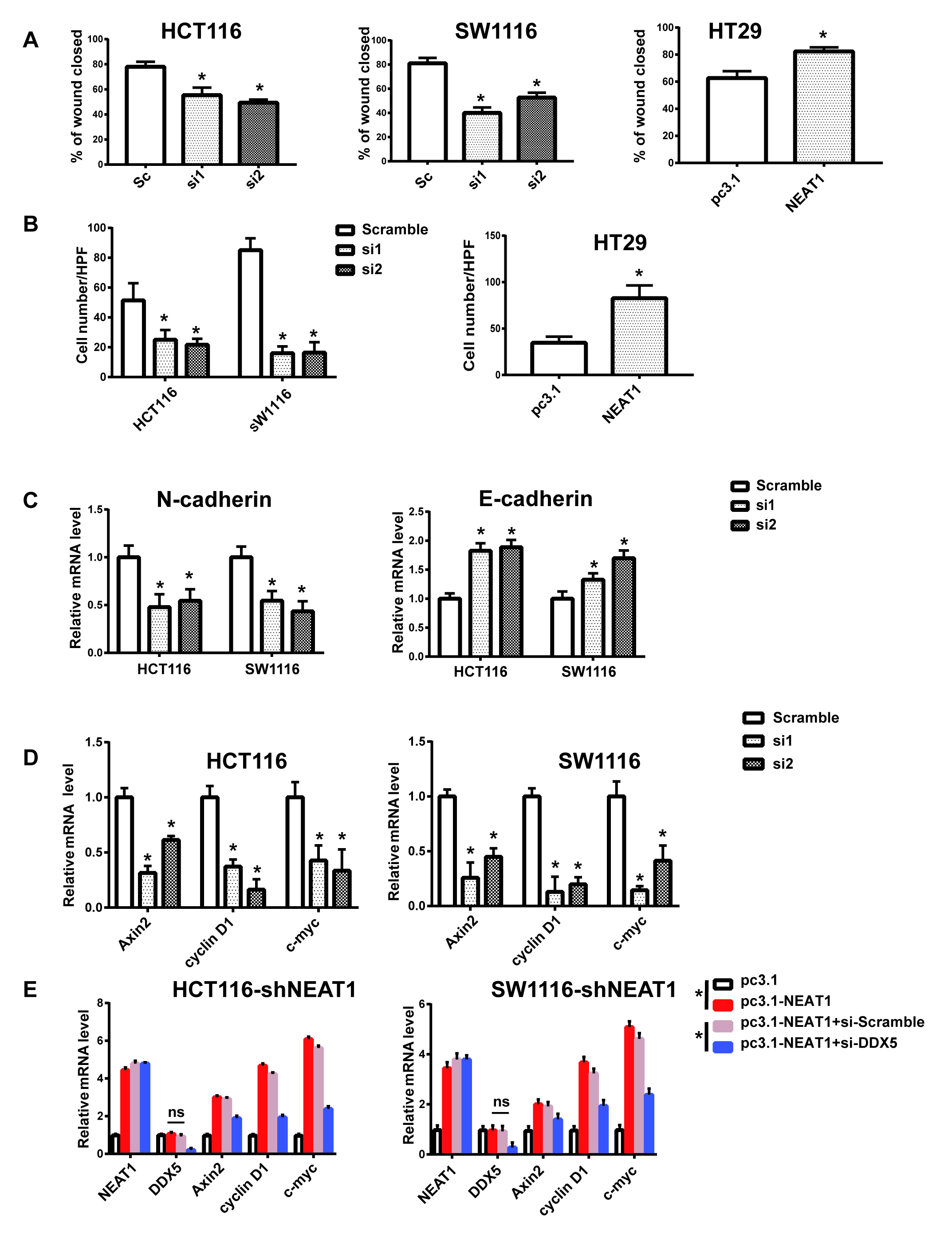

Supplement: Supplementary file 5 — Figure S3. Quantitative results for wound healing assays (A) and Transwell assays (B) of indicated CRC cells. (C) The mRNA level of N-cadherin and E-cadherin (*p < 0.05). (D) The mRNA level of Axin2, cyclin D1, and c-myc for indicated cells (*p < 0.05). (E) The mRNA level changes of DDX5, Axin2, cyclin D1, and c-myc after NEAT1 expression rescued in shNEAT1 stable cells with or without si-DDX5(*p < 0.05). (Sc represents scramble) (TIF 986 kb) [file 13045_2018_656_MOESM5_ESM.tif]
